# The genome sequence of the scotch argus butterfly, *Erebia aethiops* (Esper, 1777)

**DOI:** 10.12688/wellcomeopenres.17927.1

**Published:** 2022-08-19

**Authors:** Oskar Lohse, Konrad Lohse, Wellcome Sanger Institute Tree of Life programme, Wellcome Sanger Institute Scientific Operations: DNA Pipelines collective, Tree of Life Core Informatics collective, Hannah Augustijnen, Kay Lucek, Darwin Tree of Life Consortium

**Affiliations:** 1 Independent collector, Edinburgh, UK; 2 Institute of Evolutionary Biology, University of Edinburgh, Edinburgh, UK; 3 Department of Environmental Sciences, University of Basel, Basel, Switzerland

**Keywords:** Erebia aethiops, scotch argus, genome sequence, chromosomal, Lepidoptera

## Abstract

We present a genome assembly from an individual female *Erebia aethiops* (the scotch argus; Arthropoda; Insecta; Lepidoptera; Nymphalidae). The genome sequence is 473 megabases in span. The complete assembly is scaffolded into 20 chromosomal pseudomolecules, with the W and Z sex chromosomes assembled. The complete mitochondrial genome was also assembled and is 15.2 kilobases in length.

## Species taxonomy

Eukaryota; Metazoa; Ecdysozoa; Arthropoda; Hexapoda; Insecta; Pterygota; Neoptera; Endopterygota; Lepidoptera; Glossata; Ditrysia; Papilionoidea; Nymphalidae; Satyrinae; Erebiini; Erebia; Erebia aethiops (Esper, 1777) (NCBI:txid447833).

## Background

The Scotch argus, *Erebia aethiops* (Esper, 1777) has a wide distribution in the Palaearctic from Scotland to western Siberia and the Altai Mountains ( Wendt *et al.*, 2021). Unlike most other *Erebia* species, *E. aethiops* occurs in the lowland and montane zone. The species was first described from Scotland as the subspecies *E. aethiops caledonia* ( Newland, 2012), though this taxonomy now refers only to populations in the west and southwest of Scotland ( Newland, 2012; Thomson, 1980). Populations in the north and southeast of Scotland belong to the nominate subspecies *E. aethiops aethiops* ( Thomas & Lewington, 2016). While the two subspecies differ in their larval foodplant preference and wing morphology, with *caledonia* having narrower forewings and a narrower orange band, their taxonomic status remains disputed ( Kirkland, 1995).

In general, *E. aethiops* prefers meadows near forested areas and open woodlands ( Loertscher, 1991); ( Slamova *et al.*, 2011; Wendt *et al.*, 2021) with evidence for sex-specific preference in meso- and microhabitat use ( Slamova *et al.*, 2011; Slamova *et al.*, 2013). *E. aethiops* is univoltine, with hibernating larvae and an adult flight period from mid-July to mid-August. Larvae feed on a wide range of grasses, including *Bromus erectus*, *Brachypodium pinnatum* and, in the UK, *Molinia caerulea* and *Sesleria caerulea* ( Slamova *et al.*, 2013; Thomas & Lewington, 2016). The species may be vulnerable to anthropogenic habitat fragmentation ( Slamova *et al.*, 2013; Wendt *et al.*, 2021). Although UK populations have seen declines and northward range shifts over the last decades ( Franco *et al.*, 2006) and *E. aethiops* is now listed as Vulnerable on the UK Red List ( Fox *et al.*, 2022), it is listed as a species of Least Concern on the IUCN Red List of Europe ( van Swaay *et al.*, 2010). The karyotype of *E. aethiops* was first described as consisting of 21 chromosomes based on a single individual from Croatia ( Lorković, 1941). Although we do not know whether this chromosome count included a W, it is inconsistent with the 20 chromosomal scaffolds of this assembly ( Table 2).

## Genome sequence report

**Figure 1.**
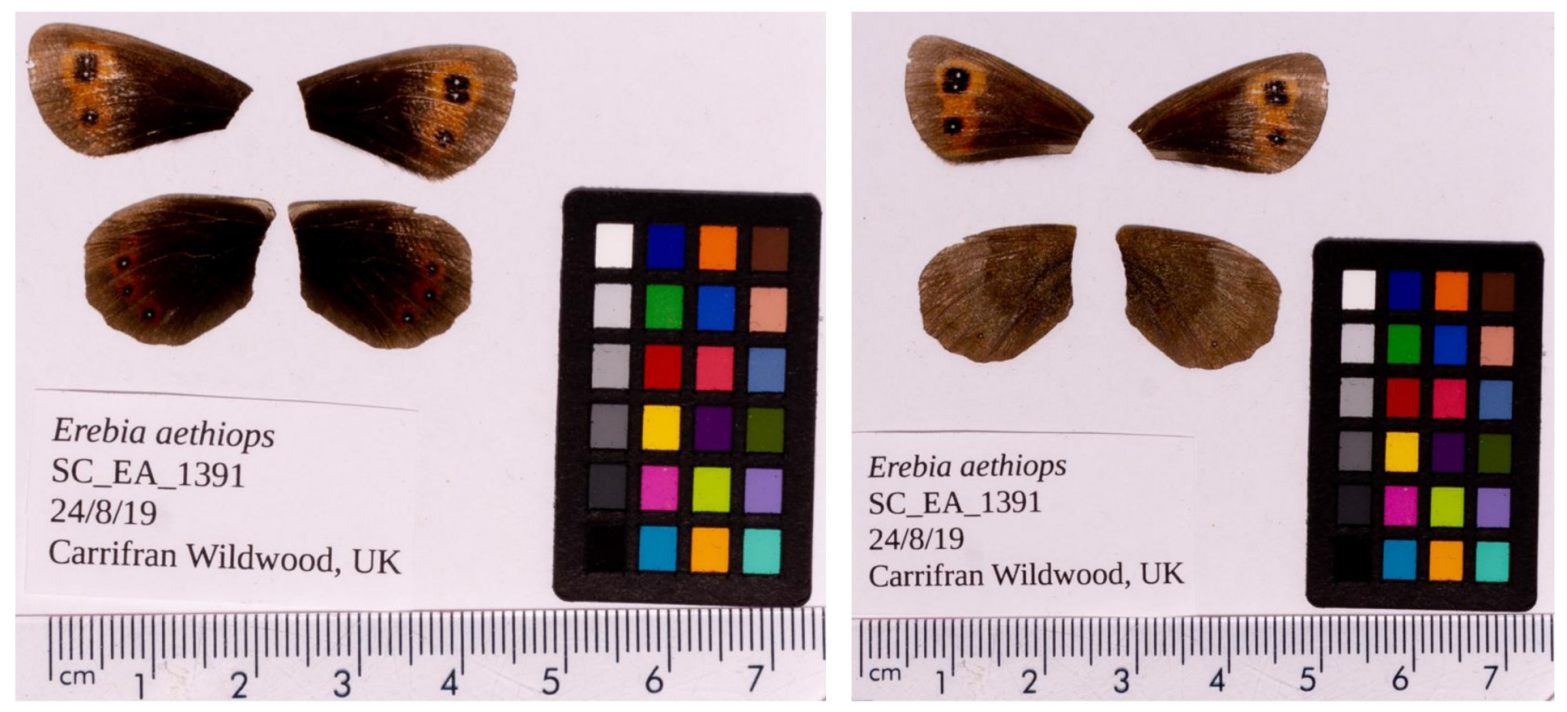
Fore and hind wings of the *Erebia aethiops* specimen from which the genome was sequenced. Dorsal (left) and ventral (right) surface views of wings from the specimen SC_EA_1391 (ilEreAeth2) from Carrifran Wildwood, Scotland, used to generate Pacific BioSciences and 10X genomics data.

The genome was sequenced from a single female *E. aethiops* ( Figure 1) collected from Carrifran Wildwood, Scotland (latitude 55.4001, longitude -3.3352). A total of 35-fold coverage in Pacific Biosciences single-molecule circular consensus (HiFi) long reads and 61-fold coverage in 10X Genomics read clouds were generated. Primary assembly contigs were scaffolded with chromosome conformation Hi-C data. Manual assembly curation corrected 30 missing/misjoins and removed two haplotypic duplications, reducing the assembly size by 0.04% and the scaffold number by 23.94%, and increasing the scaffold N50 by 8.42%.

**Figure 2.**
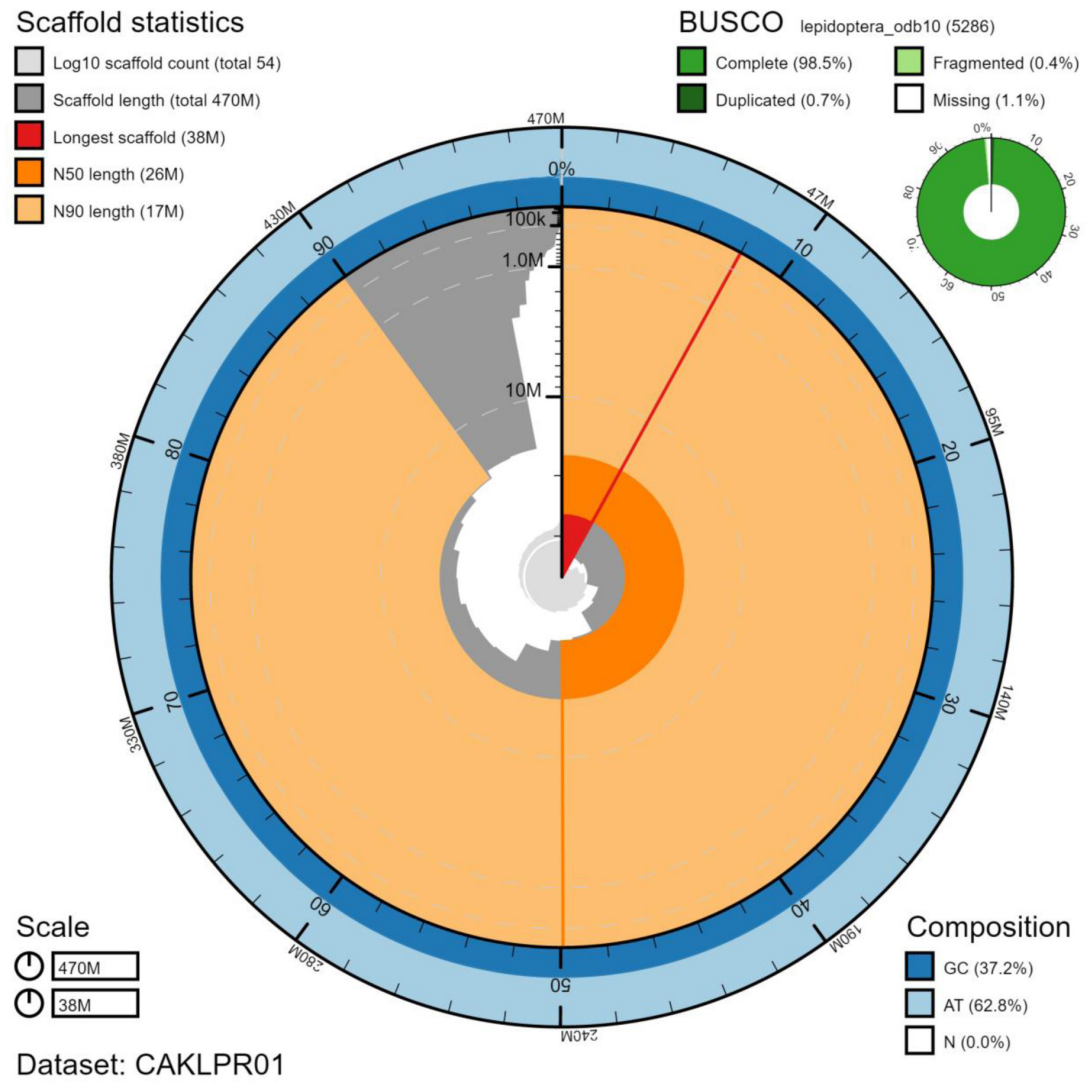
Genome assembly of *Erebia aethiops*, ilEreAeth2.1: metrics. The BlobToolKit Snailplot shows N50 metrics and BUSCO gene completeness. The main plot is divided into 1,000 size-ordered bins around the circumference with each bin representing 0.1% of the 473,469,105 bp assembly. The distribution of chromosome lengths is shown in dark grey with the plot radius scaled to the longest chromosome present in the assembly (37,954,409 bp, shown in red). Orange and pale-orange arcs show the N50 and N90 chromosome lengths (25,856,419 and 17,052,335 bp), respectively. The pale grey spiral shows the cumulative chromosome count on a log scale with white scale lines showing successive orders of magnitude. The blue and pale-blue area around the outside of the plot shows the distribution of GC, AT and N percentages in the same bins as the inner plot. A summary of complete, fragmented, duplicated and missing BUSCO genes in the lepidoptera_odb10 set is shown in the top right. An interactive version of this figure is available at https://blobtoolkit.genomehubs.org/view/ilEreAeth2.1/dataset/CAKLPR01/snail.

The final assembly has a total length of 473 Mb in 54 sequence scaffolds with a scaffold N50 of 25.9 Mb ( Table 1). The complete assembly sequence was assigned to 20 chromosomal-level scaffolds, representing 18 autosomes (numbered by sequence length), and the W and Z sex chromosomes ( Figure 2– Figure 5; Table 2). The assembly has a BUSCO v5.1.2 ( Manni *et al.*, 2021) completeness of 98.5% (single 97.8% duplicated 0.7%) using the lepidoptera_odb10 reference set (n=5,286). While not fully phased, the assembly deposited is of one haplotype. Contigs corresponding to the second haplotype have also been deposited.

**Table 1.** Genome data for *Erebia aethiops*, ilEreAeth2.1.

*Project accession data*	
Assembly identifier	ilEreAeth2.1
Species	*Erebia aethiops*
Specimen	ilEreAeth2 (genome assembly); ilEreAeth1 (additional HiFi,10X reads); ilEreAeth3 (Hi-C)
NCBI taxonomy ID	447833
BioProject	PRJEB47324
BioSample ID	SAMEA7523289
Isolate information	Female, whole organisms (ilEreAeth2, ilEreAeth1); male, whole organism (ilEreAeth3)
*Raw data accessions*	
PacificBiosciences SEQUEL II	ERR6808048 (ilEreAeth2); ERR6636094-ERR6636096, ERR6808047 (ilEreAeth1)
10X Genomics Illumina	ERR6688769-ERR6688772 (ilEreAeth2); ERR6688764-ERR6688767 (ilEreAeth1)
Hi-C Illumina	ERR6688768 (ilEreAeth3)
*Genome assembly*	
Assembly accession	GCA_923060345.1
*Accession of alternate haplotype*	GCA_923062935.1
Span (Mb)	473
Number of contigs	80
Contig N50 length (Mb)	21.4
Number of scaffolds	54
Scaffold N50 length (Mb)	25.9
Longest scaffold (Mb)	33.25
BUSCO * genome score	C:98.5%[S:97.8%,D:0.7%],F:0.4%,M:1.1%,n:5286

*BUSCO scores based on the lepidoptera_odb10 BUSCO set using v5.1.2. C= complete [S= single copy, D=duplicated], F=fragmented, M=missing, n=number of orthologues in comparison. A full set of BUSCO scores is available at https://blobtoolkit.genomehubs.org/view/ilEreAeth2.1/dataset/CAKLPR01/busco.

**Figure 3.**
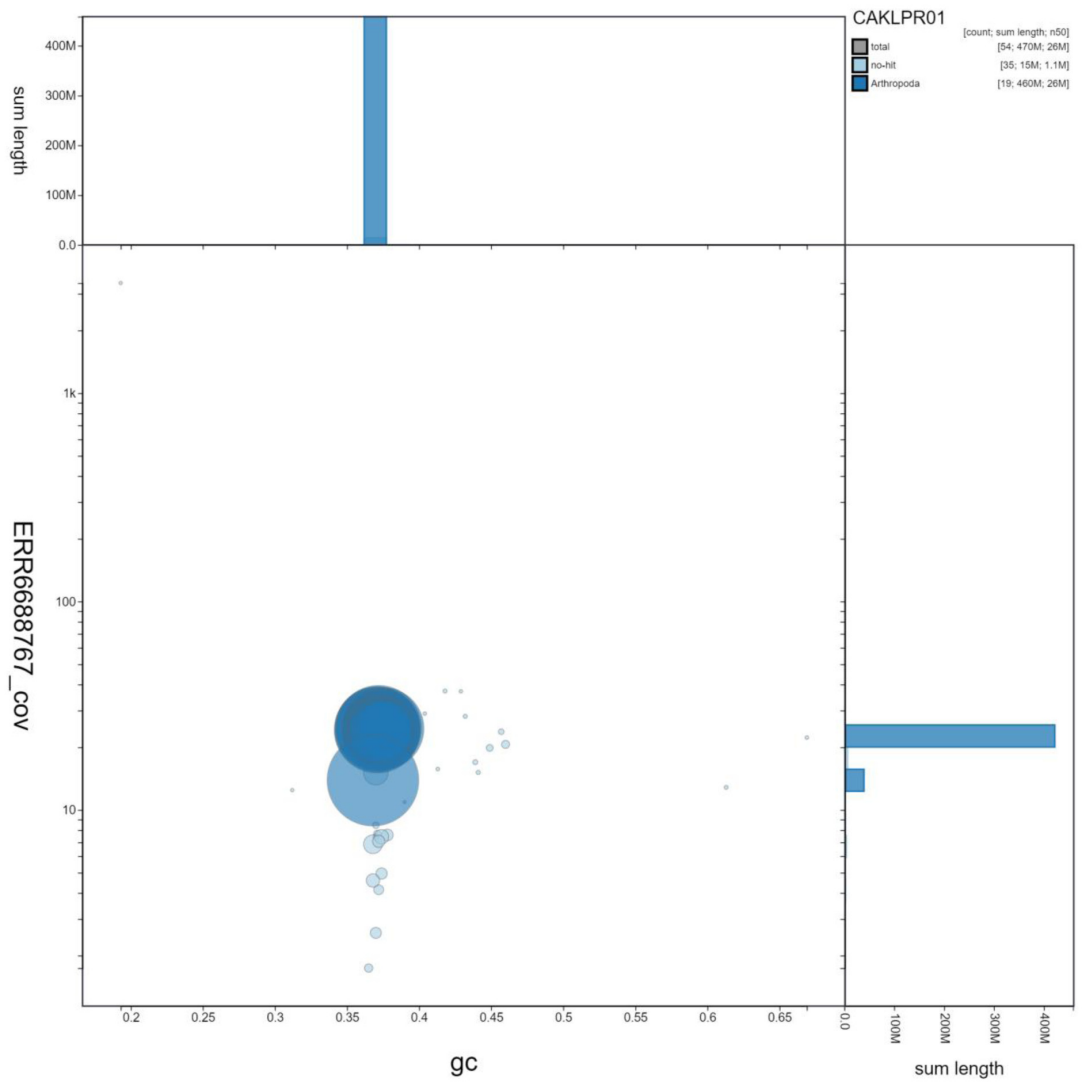
Genome assembly of *Erebia aethiops*, ilEreAeth2.1: GC coverage. BlobToolKit GC-coverage plot. Scaffolds are coloured by phylum. Circles are sized in proportion to scaffold length. Histograms show the distribution of scaffold length sum along each axis. An interactive version of this figure is available at https://blobtoolkit.genomehubs.org/view/ilEreAeth2.1/dataset/CAKLPR01/blob.

**Figure 4.**
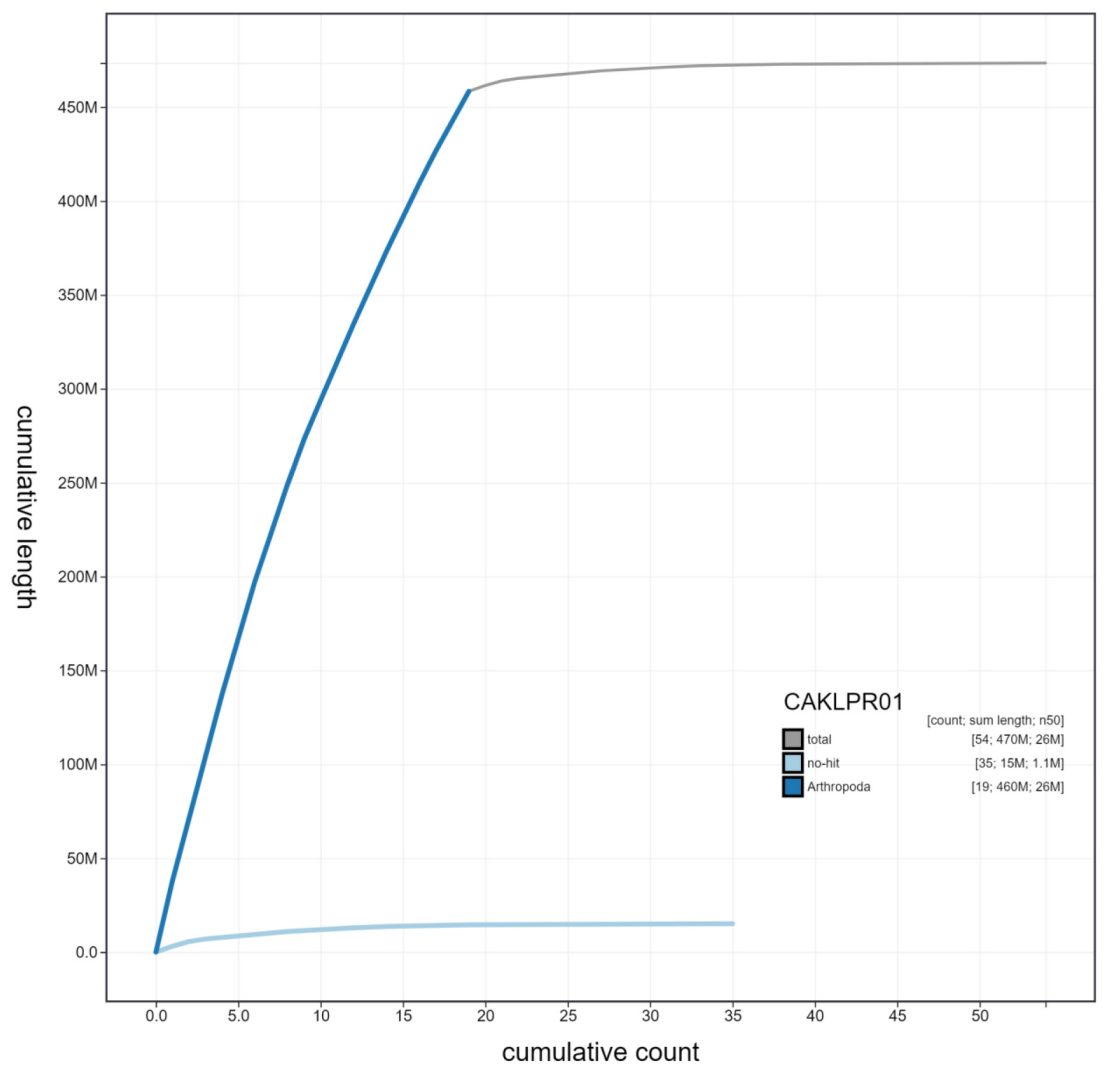
Genome assembly of *Erebia aethiops,* ilEreAeth2.1: cumulative sequence. BlobToolKit cumulative sequence plot. The grey line shows cumulative length for all scaffolds. Coloured lines show cumulative lengths of scaffolds assigned to each phylum using the buscogenes taxrule. An interactive version of this figure is available at https://blobtoolkit.genomehubs.org/view/ilEreAeth2.1/dataset/CAKLPR01/cumulative.

**Figure 5.**
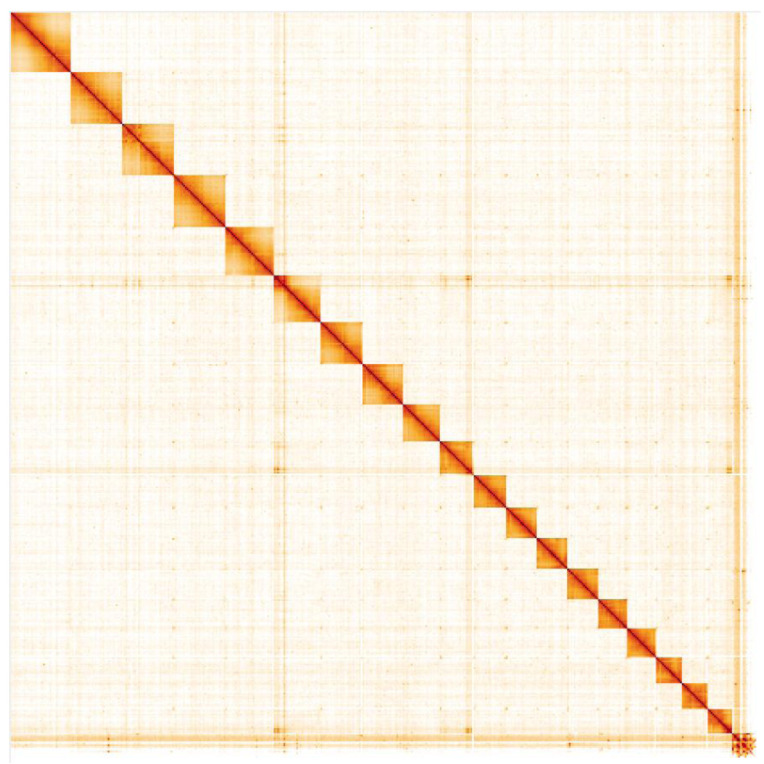
Genome assembly of *Erebia aethiops*, ilEreAeth2.1: Hi-C contact map. Hi-C contact map of the ilEreAeth2.1 assembly, visualised in HiGlass. Chromosomes are arranged in size order from left to right and top to bottom. The interactive Hi-C map can be viewed at https://genome-note-higlass.tol.sanger.ac.uk/l/?d=Es29fT2jTLK_QFHleOj4jQ.

**Table 2.** Chromosomal pseudomolecules in the genome assembly of *Erebia aethiops*, ilEreAeth2.1.

**INSDC accession**	**Chromosome**	**Size (Mb)**	**GC%**
OV281080.1	1	33.25	37.1
OV281081.1	2	32.76	37.1
OV281082.1	3	32.72	37.2
OV281083.1	4	30.44	37.1
OV281084.1	5	30.01	37.5
OV281085.1	6	26.26	37.4
OV281086.1	7	25.86	37.4
OV281087.1	8	23.96	37.2
OV281088.1	9	20.72	37.3
OV281089.1	10	20.45	37.1
OV281090.1	11	20.15	37.3
OV281091.1	12	19.45	37.3
OV281092.1	13	19.3	37.3
OV281093.1	14	18.42	37.2
OV281094.1	15	17.95	37.3
OV281095.1	16	17.05	37.3
OV281096.1	17	15.92	37.4
OV281097.1	18	15.76	37.7
OV281098.1	W	3.11	37.7
OV281079.1	Z	37.95	36.8
OV281099.1	MT	0.02	19.6
-	Unplaced	11.97	37.5

## Methods

### Sample acquisition and nucleic acid extraction

Two female *E. aethiops* specimens (ilEreAeth2, genome assembly; ilEreAeth1, additional HiFi and 10X reads) and a male (ilEreAeth3, Hi-C) were collected from Carrifran Wildwood, Scotland (latitude 55.4001, longitude -3.3352) using a net by Oskar and Konrad Lohse, who also identified the samples. Specimens were snap-frozen at -80°C.

DNA was extracted in the Tree of Life Laboratory at the Wellcome Sanger Institute. Whole organism tissue of ilEreAeth2 and ilEreAeth1 was cryogenically disrupted to a fine powder using a Covaris cryoPREP Automated Dry Pulveriser, receiving multiple impacts. Fragment size analysis of 0.01–0.5 ng of DNA was then performed using an Agilent FemtoPulse. High molecular weight (HMW) DNA was extracted using the Qiagen MagAttract HMW DNA extraction kit. Low molecular weight DNA was removed from a 200-ng aliquot of extracted DNA using 0.8X AMpure XP purification kit prior to 10X Chromium sequencing; a minimum of 50 ng DNA was submitted for 10X sequencing. HMW DNA was sheared into an average fragment size between 12–20 kb in a Megaruptor 3 system with speed setting 30. Sheared DNA was purified by solid-phase reversible immobilisation using AMPure PB beads with a 1.8X ratio of beads to sample to remove the shorter fragments and concentrate the DNA sample. The concentration of the sheared and purified DNA was assessed using a Nanodrop spectrophotometer and Qubit Fluorometer and Qubit dsDNA High Sensitivity Assay kit. Fragment size distribution was evaluated by running the sample on the FemtoPulse system.

### Sequencing

Pacific Biosciences HiFi circular consensus and 10X Genomics read cloud DNA sequencing libraries were constructed for ilEreAeth2 and ilEreAeth1 according to the manufacturers’ instructions. DNA sequencing was performed by the Scientific Operations core at the WSI on Pacific Biosciences SEQUEL II, Illumina HiSeq X (ilEreAeth1, 10X) and Illumina NovaSeq 6000 (ilEreAeth2, 10X) instruments. Hi-C data were also generated from remaining whole organism tissue of ilEreAeth3 using the Arima v2 Hi-C kit and sequenced on an Illumina NovaSeq 6000 instrument.

### Genome assembly

Assembly was carried out with Hifiasm ( Cheng *et al.*, 2021); haplotypic duplication was identified and removed with purge_dups ( Guan *et al.*, 2020). One round of polishing was performed by aligning 10X Genomics read data to the assembly with longranger align, calling variants with freebayes ( Garrison & Marth, 2012). The assembly was then scaffolded with Hi-C data ( Rao *et al.*, 2014) using SALSA2 ( Ghurye *et al.*, 2019). The assembly was checked for contamination and corrected using the gEVAL system ( Chow *et al.*, 2016) as described previously ( Howe *et al.*, 2021). Manual curation ( Howe *et al.*, 2021) was performed using gEVAL, HiGlass ( Kerpedjiev *et al.*, 2018) and Pretext. The mitochondrial genome was assembled using MitoHiFi ( Uliano-Silva *et al.*, 2021), which performs annotation using MitoFinder ( Allio *et al.*, 2020). The genome was analysed and BUSCO scores generated within the BlobToolKit environment ( Challis *et al.*, 2020). Table 3 contains a list of all software tool versions used, where appropriate.

**Table 3.** Software tools used.

**Software tool**	**Version**	**Source**
Hifiasm	0.15.3-r339	Cheng *et al.*, 2021
purge_dups	1.2.3	Guan *et al.*, 2020
SALSA2	2.2	Ghurye *et al.*, 2019
longranger align	2.2.2	https://support.10xgenomics.com/genome-exome/ software/pipelines/latest/advanced/other-pipelines
freebayes	1.3.1-17- gaa2ace8	Garrison & Marth, 2012
MitoHiFi	2.0	Uliano-Silva *et al.*, 2021
HiGlass	1.11.6	Kerpedjiev *et al.*, 2018
PretextView	0.2.x	https://github.com/wtsi-hpag/PretextView
BlobToolKit	3.0.5	Challis *et al.*, 2020

## Data availability

European Nucleotide Archive: Erebia aethiops (Scotch argus), Accession number PRJEB47324; https://identifiers.org/ena.embl/PRJEB47324.

The genome sequence is released openly for reuse. The *E. aethiops* genome sequencing initiative is part of the Darwin Tree of Life (DToL) project. The genome will be annotated and presented through the Ensembl pipeline at the European Bioinformatics Institute. Raw data and assembly accession identifiers are reported in Table 1.

## Author information

Members of the Wellcome Sanger Institute Tree of Life programme are listed here: https://doi.org/10.5281/zenodo.6125027.

Members of Wellcome Sanger Institute Scientific Operations: DNA Pipelines collective are listed here: https://doi.org/10.5281/zenodo.5746904.

Members of the Tree of Life Core Informatics collective are listed here: https://doi.org/10.5281/zenodo.6125046.

Members of the Darwin Tree of Life Consortium are listed here: https://doi.org/10.5281/zenodo.5638618.
