# Supplementary material for: The genome sequence of the scotch argus butterfly, Erebia aethiops (Esper, 1777)
Source: Wellcome Open Res. 2022 Aug 19;7:217. [Version 1] doi: 10.12688/wellcomeopenres.17927.1 (PMC9445563; doi:10.12688/wellcomeopenres.17927.1)
Supplement: Peer review history

# Peer review: Reviewer response for version 1

**DOI:** 10.21956/wellcomeopenres.19865.r52117

**Published:** 2022-09-05

**Authors:** Jing Zhang

**Affiliations:** 1 Eugene McDermott Center for Human Growth and Development, University of Texas Southwestern Medical Center, Dallas, TX, USA

Authors present the high-quality chromosome-level genome assembly for *Erebia aethiops. *The assembly includes W chromosome which is rarely included in other Lepidoptera genomes. This high quality assembly will benefit future studies of *E. aethiops* and other related insects. The assembly pipeline is described in detail and also repeated by the Darwin Tree of Life (DToL) project for numerous other high quality assemblies too.

Are sufficient details of methods and materials provided to allow replication by others?

Yes

Is the rationale for creating the dataset(s) clearly described?

Yes

Are the datasets clearly presented in a useable and accessible format?

Yes

Are the protocols appropriate and is the work technically sound?

Yes

Reviewer Expertise:

Genomics, Computational biology, bioinformatics

I confirm that I have read this submission and believe that I have an appropriate level of expertise to confirm that it is of an acceptable scientific standard.

# Peer review: Reviewer response for version 1

**DOI:** 10.21956/wellcomeopenres.19865.r52112

**Published:** 2022-08-24

**Authors:** Wei Zhang

**Affiliations:** 1 State Key Laboratory of Protein and Plant Gene Research, Peking-Tsinghua Center for Life Sciences, Academy for Advanced Interdisciplinary Studies, School of Life Sciences, Peking University, Beijing, China

Lohse *et al.* report a chromosome-level genome assembly of the scotch argus butterflies with both W and Z chromosomes assembled. This genome assembly has a considerable N50 value and good BUSCO statistics. The methods section is well written as well. I think the overall quality of this genome assembly is valid and will benefit relevant studies. Therefore, I recommend this manuscript be accepted for indexing.

Are sufficient details of methods and materials provided to allow replication by others?

Yes

Is the rationale for creating the dataset(s) clearly described?

Yes

Are the datasets clearly presented in a useable and accessible format?

Yes

Are the protocols appropriate and is the work technically sound?

Yes

Reviewer Expertise:

evolutionary genomics

I confirm that I have read this submission and believe that I have an appropriate level of expertise to confirm that it is of an acceptable scientific standard.
